# Optimizing EDM of Gunmetal with Al_2_O_3_-Enhanced Dielectric: Experimental Insights and Machine Learning Models

**DOI:** 10.3390/ma18194578

**Published:** 2025-10-02

**Authors:** Saumya Kanwal, Usha Sharma, Saurabh Chauhan, Anuj Kumar Sharma, Jitendra Kumar Katiyar, Rabesh Kumar Singh, Shalini Mohanty

**Affiliations:** 1 Centre for Advanced Studies, Lucknow 226031, Uttar Pradesh, India; saumyakanwal1999@gmail.com (S.K.); anujksharma@cas.res.in (A.K.S.); 2 Department of Information Technology, Babu Banarasi Das Institute of Technology and Management, Lucknow 226028, Uttar Pradesh, India; 3 Applied Science and Humanities Department, Rajkiya Engineering College, Kannauj 209732, Uttar Pradesh, India; saurabhchauhan09@gmail.com; 4 Department of Mechanical and Industrial Engineering, Manipal Institute of Technology, Manipal Academy of Higher Education, Manipal 576104, Karnataka, India; 5 Mechanical Engineering Department, Madan Mohan Malaviya University of Technology, Gorakhpur 273010, Uttar Pradesh, India; 6 Faculty of Engineering and Sciences, University of Greenwich, Chatham Maritime ME4 4TB, UK

**Keywords:** EDM, gunmetal, Al_2_O_3_ nanoparticles, machine learning, Taguchi design, surface roughness, tool wear rate, ANOVA

## Abstract

This study investigates the optimization of electric discharge machining (EDM) parameters for gunmetal using copper electrodes in two different dielectric environments, which are conventional EDM oil and EDM oil infused with Al_2_O_3_ nanoparticles. A Taguchi L27 orthogonal array design was used to evaluate the effects of current, voltage, and pulse-on time on Material Removal Rate (MRR), Electrode Wear Rate (EWR), and surface roughness (Ra, Rq, and Rz). Analysis of Variance (ANOVA) was used to statistically evaluate the influence of each parameter on machining performance. In addition, machine learning models including Linear Regression, Ridge Regression, Support Vector Regression, Random Forest, Gradient Boosting, and Neural Networks were implemented to predict performance outcomes. The originality of this research is not only rooted in the introduction of new models; rather, it is also found in the comparative analysis of various machine learning methodologies applied to the performance of electrical discharge machining (EDM) utilizing Al_2_O_3_-enhanced dielectrics. This investigation focuses specifically on gunmetal, a material that has not been extensively studied within this framework. The nanoparticle-enhanced dielectric demonstrated improved machining performance, achieving approximately 15% higher MRR, 20% lower EWR, and 10% improved surface finish compared to conventional EDM oil. Neural Networks consistently outperformed other models in predictive accuracy. Results indicate that the use of nanoparticle-infused dielectrics in EDM, coupled with data-driven optimization techniques, enhances productivity, tool life, and surface quality.

## 1. Introduction

Electric discharge machining (EDM) is a widely adopted non-traditional machining process used to fabricate intricate and high-precision components, particularly from difficult-to-machine materials [1]. Unlike conventional methods, EDM operates through a series of electrical discharges between a tool and workpiece, both submerged in a dielectric fluid [2,3,4]. The dielectric fluid acts as an insulator until breakdown voltage is reached, facilitating controlled spark erosion [5,6,7]. Over the years, a considerable amount of research has been conducted to optimize various aspects of the EDM process, including electrode materials, dielectric fluids, and machining parameters. Among the critical process variables that influence EDM performance, there are the dielectric fluid properties, machining parameters, and electrode–workpiece material pairing [8,9,10].

Traditionally, hydrocarbon-based dielectric oils have been the preferred choice, given their high breakdown voltage and effective flushing capabilities [11]. However, these conventional dielectrics also present drawbacks, including high costs, environmental concerns, and the need for frequent fluid replacement [4]. This has motivated researchers to explore alternative dielectric solutions that could offer improved performance, lower environmental impact, and reduced operational costs [12,13,14,15]. Recent trends in dielectric modification include powder-mixed EDM, nanoparticle-infused dielectrics, gaseous dielectrics, and cryogenic or hybrid treatments [16,17,18]. While many of these studies are devoted to machining materials with limited electrical conductivity, their theoretical frameworks are also applicable to conductive alloys [19].

One of the most promising developments in this area is the use of nanoparticle-infused dielectrics [20]. These advanced dielectric fluids, which incorporate nanoparticles like Al_2_O_3_, have been shown to enhance the EDM process by improving electrical conductivity and thermal properties [21,22]. This, in turn, can lead to increased MRR, reduced EWR, and improved surface finish [23,24]. Despite these advancements, there is still a need for more comprehensive studies that compare the performance of these alternative dielectrics under varying machining conditions [25]. In particular, the interaction between specific tool–workpiece combinations and dielectric fluids remains underexplored.

Another critical factor influencing EDM performance is the flushing of the machining gap, which ensures debris removal and stable discharge conditions [26]. Various methods such as jet flushing, side flushing, suction flushing, and rotary flushing are used in EDM practice [27,28,29]. In the present study, direct nozzle flushing was employed in the tool–workpiece gap to maintain a stable machining zone. The dielectric fluid temperature was maintained at room temperature.

In the literature, most of the research related to EDM parameter optimization depends on trial and error, a time-consuming and costly approach that limits machining efficiency [30]. In contrast, machine learning offers a data-driven methodology capable of identifying complex patterns and relationships between machining parameters and performance outcomes [31,32,33,34]. Moreover, the integration of machine learning into the EDM optimization process opens new possibilities for the manufacturing industry, providing a framework for future innovations [35,36].

Gunmetal, an alloy composed primarily of copper, tin, and zinc, is widely used in industries such as marine engineering and hydraulic casting due to its excellent corrosion resistance and machinability [37,38]. To address these gaps, this study systematically compares EDM performance under conventional and nanoparticle-enhanced dielectric conditions using gunmetal as the workpiece material and copper as the tool. Key response metrics including MRR, EWR, and surface roughness (Ra, Rq, Rz) are experimentally measured, and predictive modeling is carried out using various machine learning algorithms to enhance process optimization and forecasting. The novelty of this study lies in integrating Al_2_O_3_ nanoparticle-enhanced dielectrics with a comparative application of multiple machine learning models, specifically for EDM of gunmetal. This dual approach provides both new experimental insights and predictive capability, offering practical guidance for real-world EDM applications.

## 2. Materials and Methods

### 2.1. Material Selection

The choice of materials for the tool electrode and workpiece is critical in influencing the efficiency and quality of the EDM process [39]. Copper was selected as the electrode material due to its exceptional electrical conductivity (100% IACS) and high thermal conductivity (390–400 W/m·K), which enable efficient energy transfer and stable spark generation [40,41,42,43]. Its good machinability and moderate hardness also allow for fabrication into precise geometries while maintaining structural integrity during machining [44].

Gunmetal, an alloy containing approximately 88% copper, 10% tin, and 2% zinc, was used as the workpiece owing to its corrosion resistance, machinability, and moderate mechanical strength. With tensile strength ranging from 200 to 250 MPa and a thermal conductivity of 40–50 W/m·K, gunmetal withstands thermal stress and maintains dimensional stability under EDM conditions. Its industrial relevance in marine and hydraulic applications further justifies its selection for this study.

**Figure 1:**
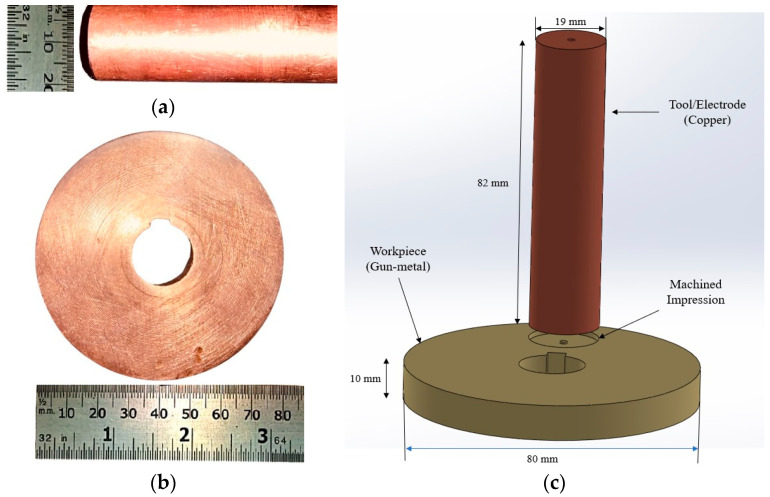
(**a**) Copper tool; (**b**) gunmetal as workpiece; (**c**) schematic diagram with dimensions of electrode and workpiece.

The copper–gunmetal pairing offers a compatible combination for EDM, with manageable electrode wear and efficient spark erosion as shown in Figure 1. Their relatively close thermal expansion coefficients and thermal properties help minimize distortion and enhance surface finish. This synergy makes the pair ideal for investigating the effects of dielectric modification. A summary of material properties and machining parameters is provided in Table 1.

### 2.2. Preparation of Al_2_O_3_ Nanoparticle-Mixed EDM Oil

To enhance the dielectric properties of conventional EDM oil, aluminum oxide (Al_2_O_3_) nanoparticles with an average particle size of 50 nm were incorporated at a concentration of 0.5 wt.%. The inclusion of these nanoparticles aimed to improve thermal conductivity, promote micro-convection through Brownian motion, and establish efficient thermal pathways during machining [45].

**Figure 2:**
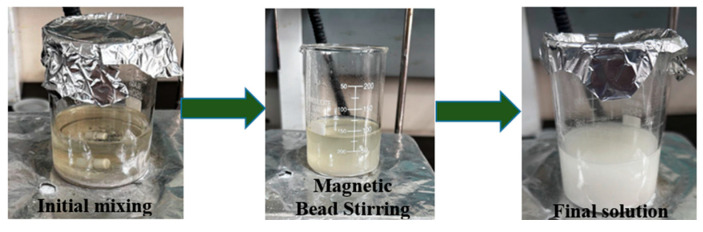
Preparation of Al_2_O_3_ nanoparticle-mixed EDM oil.

The preparation followed a two-step dispersion process (Figure 2). First, 2 g of Al_2_O_3_ nanoparticles was added to a measured volume of EDM oil and subjected to magnetic stirring using a Remi laboratory stirrer (Model: REMI 10 MLH PLUS, Mumbai, India), initiating dispersion by generating a vortex. This step was followed by ultrasonication at ~40 kHz in a Sonics Vibra-Cell bath for 12–18 h, producing cavitation and high shear forces that de-agglomerated particles and ensured homogeneous mixing. Finally, the nanoparticle-mixed dielectric was filtered to eliminate residual agglomerates and impurities. This well-established procedure facilitates consistent machining performance through improved heat dissipation, enhanced MRR, reduced EWR, and superior surface quality.

### 2.3. FTIR Spectral Analysis of EDM Oil with and Without Al_2_O_3_

**Figure 3:**
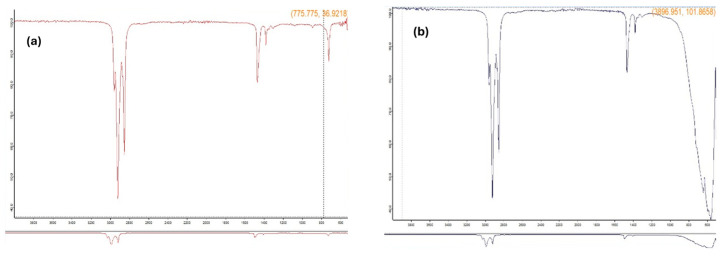
(**a**) FTIR spectrum of pure EDM oil; (**b**) Al_2_O_3_ nanoparticle-mixed EDM oil.

Fourier Transform Infrared spectroscopy was performed on (Model: FTIR spectrophotometer Alpha-II, Bruker, Chennai, India) to examine the chemical interactions induced by the addition of Al_2_O_3_ nanoparticles to EDM oil. Figure 3a shows the FTIR spectrum for pure EDM oil, which displays characteristic hydrocarbon peaks such as C-H stretching (2900–2950 cm^−1^) and C-O stretching (1000–1300 cm^−1^).

Upon adding Al_2_O_3_ nanoparticles, the FTIR spectrum (Figure 3b) exhibited new peaks in the 500–800 cm^−1^ range, corresponding to metal–oxygen (M–O) stretching, confirming the presence of Al_2_O_3_. The observed changes in peak intensity and position suggest meaningful interactions between the nanoparticles and the base fluid. Table 2 summarizes the key spectral differences. These spectral modifications support the hypothesis that Al_2_O_3_ incorporation enhances the dielectric properties, potentially leading to more stable discharges and improved machining output.

### 2.4. Equipment and Experimental Setup

**Figure 4:**
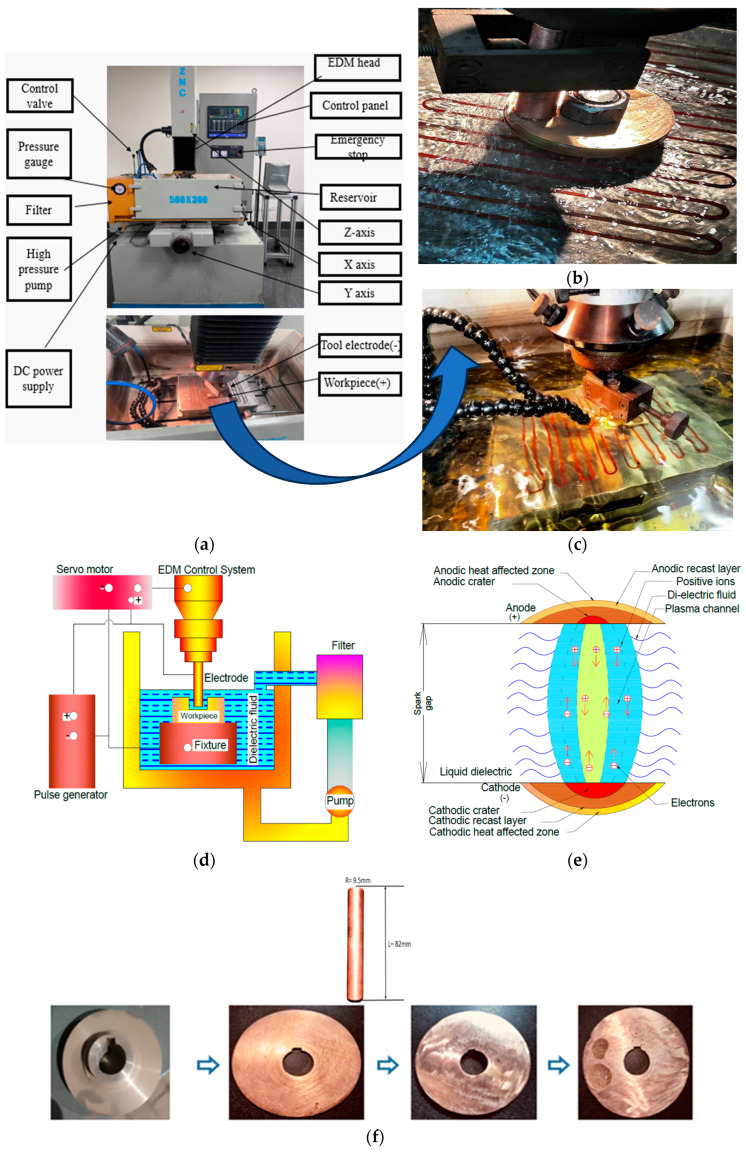
(**a**) Complete experimental setup (**b**,**c**); close view of the EDM experimental setup; (**d**) graphical illustration of the EDM process; (**e**) plasma generation during sparking; (**f**) electrode material (copper) and workpiece (gunmetal) sample preparation from waste flange coupling.

All EDM experiments were conducted on a CNC EDM machine (Model: 5030 ZNC, Make: Electronica, Munich, Germany) equipped with high-resolution control systems. The setup included a dielectric supply unit, electrode holder, and workpiece fixture. Gunmetal samples were fabricated from waste flange material and were machined flat to ensure uniform contact. Figure 4 illustrates the experimental arrangement, including detailed views of the equipment, the dielectric flow system, and the sparking zone.

A dedicated reservoir supplied either pure EDM oil or Al_2_O_3_-enhanced EDM oil as needed. To maintain dielectric integrity, the system included a filtration unit. Schematics of the EDM mechanism and real-time sparking visuals are also provided in Figure 4. This setup ensured consistency across all trials and minimized external variability. The dielectric was flushed using a direct flushing system, in which pressure was directed into the tool–workpiece gap. The flushing electrode was lifted every ~2 s for ~0.5 s to facilitate debris removal. The duty cycle (Ton/Toff) was maintained at ~0.5–0.6, while the dielectric temperature was kept constant at 27 ± 2 °C. The burn-through state duration of the electrode was consistently <1 s across all experiments.

### 2.5. Experimental Design

The experimental plan was developed using a Taguchi L27 orthogonal array to analyze the effects of machining parameters and dielectric type. The variables studied included current (5 A, 10 A, 15 A), voltage (30 V, 40 V, 50 V), and pulse-on time (30 µs, 50 µs, 75 µs). Each experiment was repeated three times to ensure statistical reliability, yielding a total of 54 trials (27 per dielectric condition).

**Figure 5:**
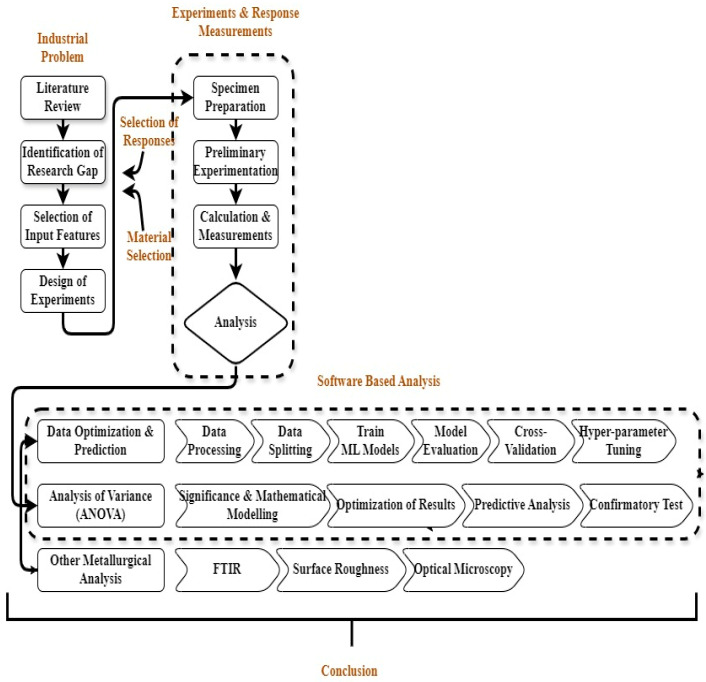
Methodology of the research work.

Design of Experiments (DOE) was created using Minitab Statistical Software (Version 21.1.0). For each test, the machine was flushed and filled with the designated dielectric fluid. The electrode and gunmetal workpiece were mounted, and machining was conducted until the specified depth was reached. After completion, the workpieces were cleaned and prepared for surface and wear analysis. A schematic overview of the experimental methodology is shown in Figure 5.

## 3. Results and Discussion

Following the design of experiments, machining was carried out using the EDM machine, and data were collected for each set of parameters. All the readings were measured five times, and averages of the values were recorded for each set of experiments. After each experiment, the machine was cleaned and reconfigured with new parameters. A total of 54 experimental runs were conducted, i.e., 27 with pure EDM oil and 27 with Al_2_O_3_-mixed EDM oil. Table 3 and Table 4 display the measured MRR, EWR, and surface roughness values (Ra, Rq, Rz) for both dielectric conditions.

### 3.1. Influence of Process Parameters on MRR and EWR

**Figure 6:**
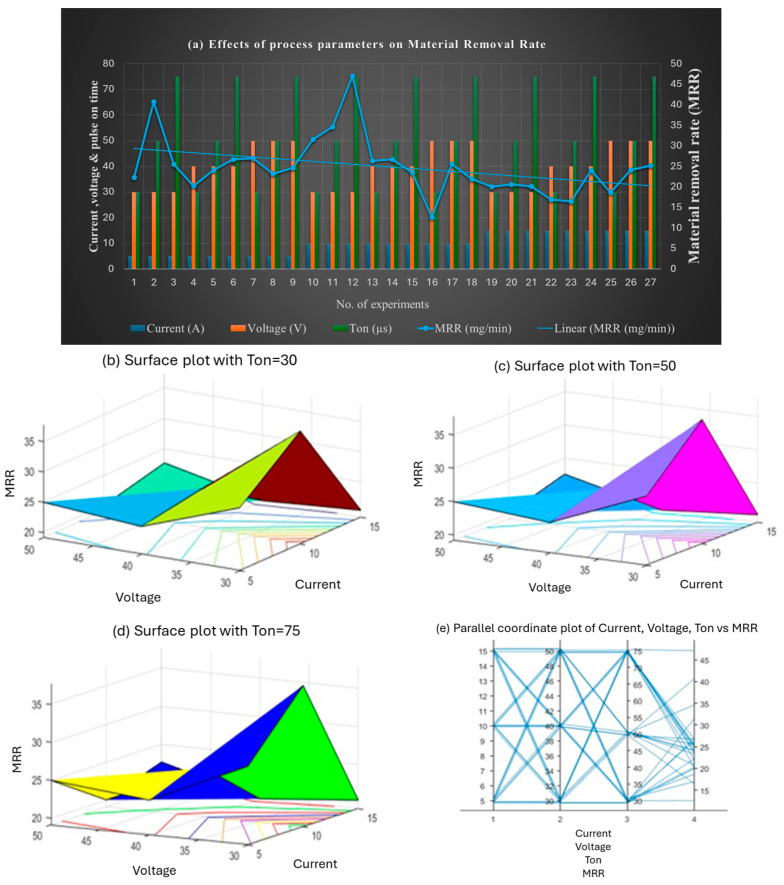
(**a**) Effect of combined set of process parameters on MRR; 3D surface plots representing the effect of voltage, current, and Ton on MRR at (**b**) Ton = 30 µs, (**c**) Ton = 50 µs, and (**d**) Ton = 75 µs; and (**e**) interaction plot.

Graphical representations were used to visualize the distribution and trends of the response variables. Figure 6 shows the influence of current (Ip), voltage (Vg), and pulse-on time (Ton) on MRR. Higher currents (e.g., 15 A) generally resulted in increased MRR. Among the factors, current and Ton had a more significant impact on MRR than voltage. Longer pulse durations, particularly at 75 µs, led to greater material removal. The trend line in the graph demonstrates this combined effect.

The highest MRR value (47.1 mg/min) was observed at 10 A, 30 V, and 75 µs, indicating that high current and extended Ton are optimal for maximizing MRR. Results indicate that the highest MRR is achieved at a combination of high current and long pulse duration during the machining of gunmetal workpieces using a copper tool in EDM. Finally, the results clearly reveal that current and pulse-on time are the primary factors that influence MRR, while voltage also plays a crucial role, but its impact on MRR is comparatively smaller.

**Figure 7:**
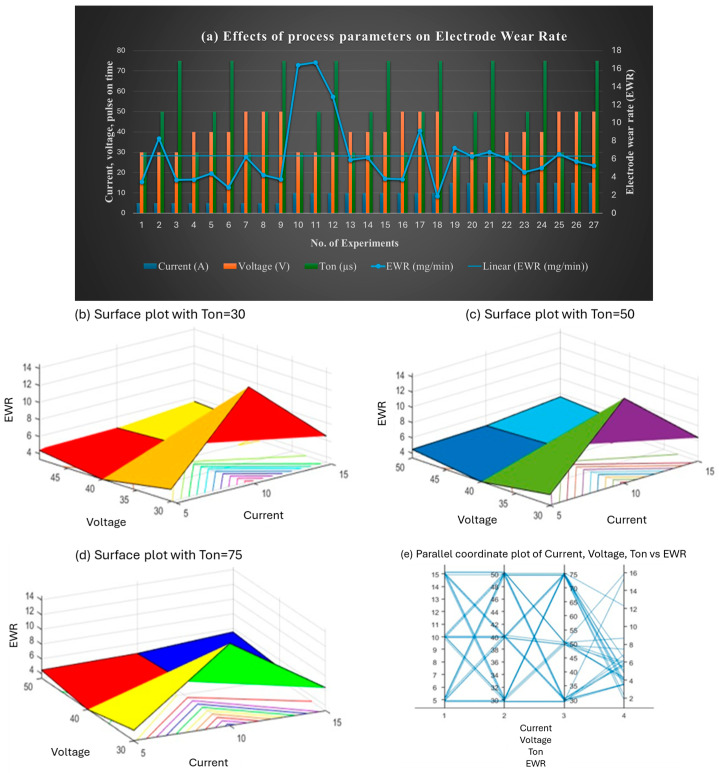
(**a**) Effect of the combined set of process parameters on EWR; 3D surface plots representing the effect of voltage, current, and Ton on EWR at (**b**) Ton = 30 µs, (**c**) Ton = 50 µs, and (**d**) Ton = 75 µs; and (**e**) interaction plot.

Figure 7 illustrates the effect of process parameters on EWR. Lower currents (e.g., 5 A) yielded lower EWR values, although exceptions occurred at specific combinations. EWR peaked at 10 A and 30 V and stabilized at higher voltages (50 V). Ton also influenced EWR, with shorter pulses (30 µs) showing higher variability and longer pulses (75 µs) contributing to stable wear rates. The findings suggest that minimizing EWR requires higher voltages and moderate pulse durations, with careful selection of current.

### 3.2. Influence on Surface Roughness (Ra, Rq, Rz)

**Figure 8:**
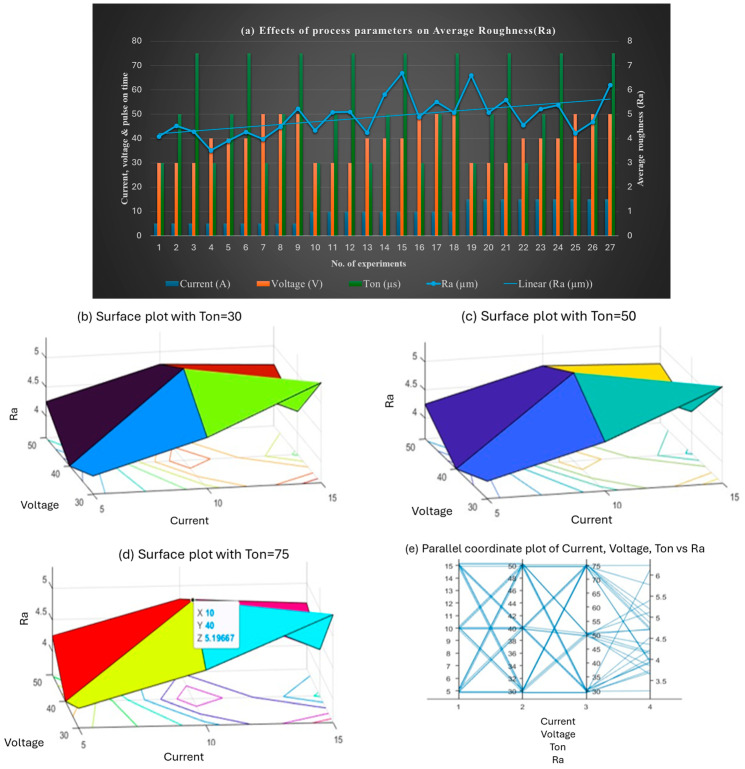
(**a**) Effect of the combined set of process parameters on average surface roughness, Ra; 3D surface plots representing the effect of voltage, current, and Ton on average surface roughness at (**b**) Ton = 30 µs, (**c**) Ton = 50 µs, and (**d**) Ton = 75 µs; and (**e**) interaction plot.

Figure 8 shows the effect of parameters on Ra. The lowest Ra values were recorded at 5 A current, with minimal changes as voltage and Ton increased slightly. The highest Ra values occurred at 15 A, especially with lower voltage and shorter Ton. Voltage had a significant effect on Ra, particularly at increased Ton values. Shorter Ton (30 µs) resulted in lower Ra, while longer Ton (75 µs) increased Ra. The optimal Ra was achieved through the effective control of current and Ton.

**Figure 9:**
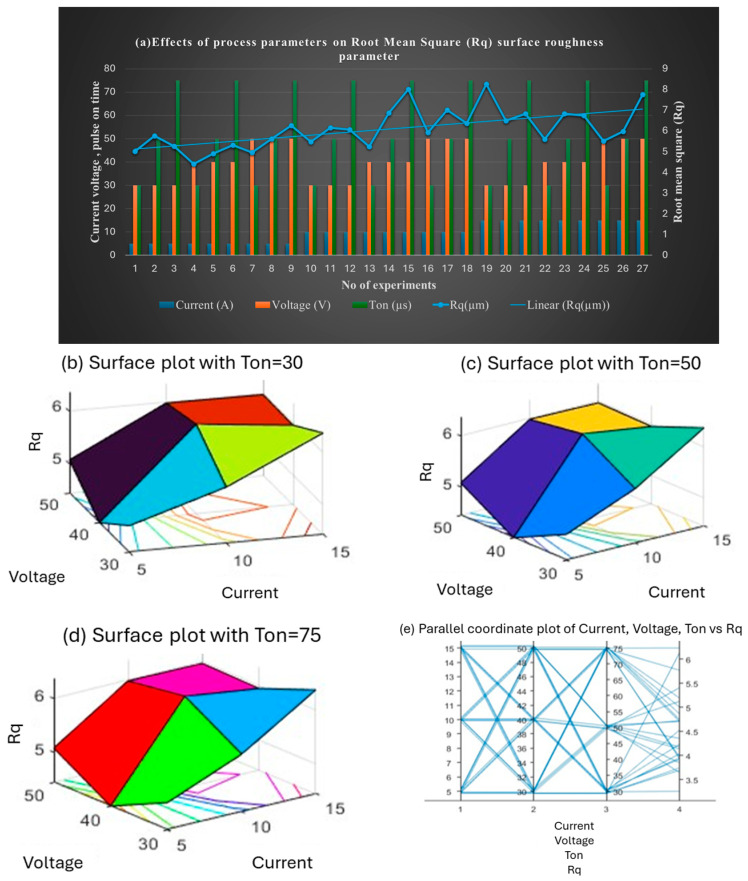
(**a**) Effect of the combined set of process parameters on root mean square parameter, Rq; 3D surface plots representing the effect of voltage, current, and Ton on Rq at (**b**) Ton = 30 µs, (**c**) Ton = 50 µs, and (**d**) Ton = 75 µs; and (**e**) interaction plot.

Figure 9 presents the impact on Rq (root mean square roughness). At 5 A, Rq values were low but rose with increases in voltage and Ton. The lowest Rq (4.41 µm) was observed at 5 A, 40 V, and 30 µs, while the highest (6.26 µm) occurred at 5 A, 50 V, and 75 µs. As current increased, Rq became more variable. The highest Rq (8.26 µm) was recorded at 15 A and 30 V with a short Ton. Ton had a significant influence on Rq, with lower values at 30 µs and higher values at 75 µs.

**Figure 10:**
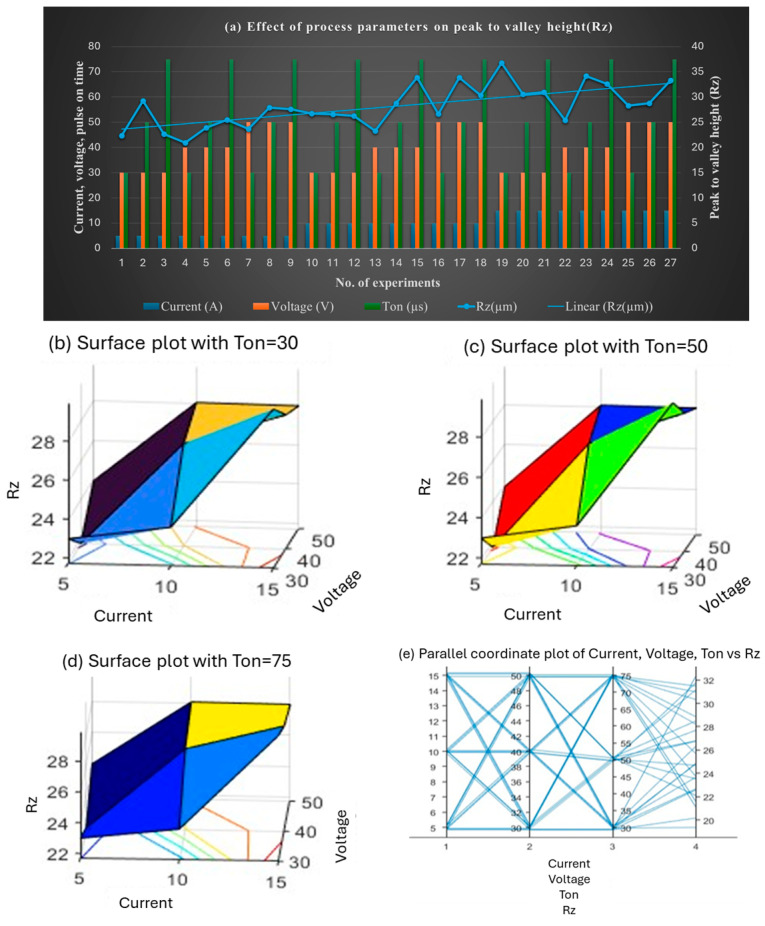
(**a**) Effect of the combined set of process parameters on mean peak-to-valley height, Rz; 3D surface plots representing the effect of voltage, current, and Ton on Rz at (**b**) Ton = 30 µs, (**c**) Ton = 50 µs, and (**d**) Ton = 75 µs; and the (**e**) interaction plot.

Figure 10 illustrates the effect on Rz (peak-to-valley height). The lowest Rz (20.93 µm) occurred at 5 A, 40 V, and 30 µs. Rz increased slightly with higher voltage and Ton, peaking at 33.86 µm for 10 A, 40 V, and 100 µs. The maximum Rz (36.78 µm) was recorded at 15 A, 30 V, and 30 µs. These results highlight the importance of selecting optimal parameters to minimize Rz.

### 3.3. Implementation of Machine Learning Models

**Figure 11:**
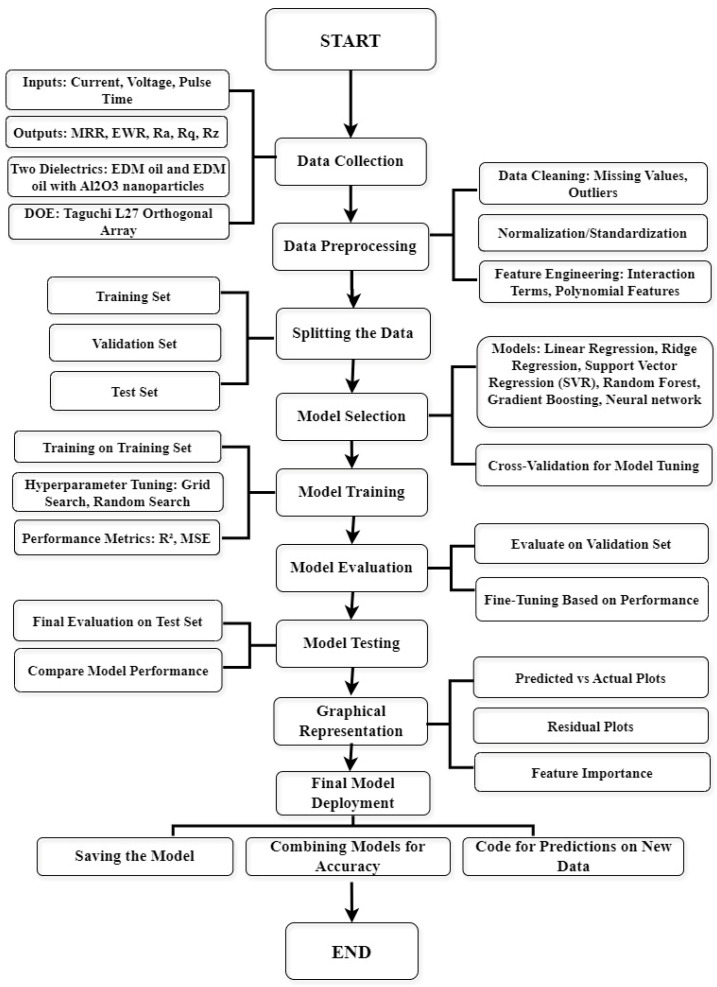
Schematic flowchart of the machine learning workflow for EDM research.

Figure 11 illustrates the comprehensive machine learning workflow adopted in this study. It starts with data collection, capturing inputs such as current, voltage, and pulse-on time, along with outputs like MRR, EWR, and surface roughness parameters (Ra, Rq, Rz).

The collected data underwent preprocessing, which included splitting into training, validation, and test sets. Various regression and ensemble models were trained and evaluated based on their performance. The final models were tested, with optional inclusion of dimensionality reduction and ensemble techniques. The entire process was visually summarized in the flowchart shown in Figure 11.

a. - Experimental Design and Data Collection

A structured design approach was implemented using the Taguchi L27 orthogonal array to explore the effects of current, voltage, and pulse-on time on machining responses. Data were collected under the following two dielectric conditions: pure EDM oil and Al_2_O_3_-mixed EDM oil. The results were compiled in a spreadsheet for analysis.

b. Data Preprocessing

To prepare the data for machine learning models, several preprocessing steps were carried out:

- Data Cleaning: Missing values and outliers were addressed.
- Normalization: Numerical features such as current, voltage, and Ton were scaled for consistency.
- Feature Engineering: The categorical variable “Dielectric Type” was one-hot encoded to differentiate the two dielectric conditions.

c. Model Selection and Training

The dataset was divided into training, validation, and test subsets. The following six machine learning models were selected to capture both linear and nonlinear patterns:

- Linear Regression;
- Ridge Regression;
- Support Vector Regression (SVR);
- Random Forest;
- Gradient Boosting;
- Neural Networks.

Each model was tuned using techniques such as grid search and random search to optimize hyperparameters. The models were evaluated using the following metrics:

- Mean Squared Error (MSE)*:* Measures the average squared difference between actual and predicted values.
- Absolute Mean Error (AME): Represents the average of absolute differences between actual and predicted values.
- Sum of Squared Errors (SSE): Sum of squared differences between actual and predicted values.
- Root Mean Squared Error (RMSE): Square root of MSE, gives an idea of the magnitude of the errors.
- Coefficient of Determination (R^2^): Indicates how well the model explains the variability of the target variable; a value closer to 1 means a better fit.

#### 3.3.1. Model Performance Metrics for MRR and EWR

**Figure 12:**
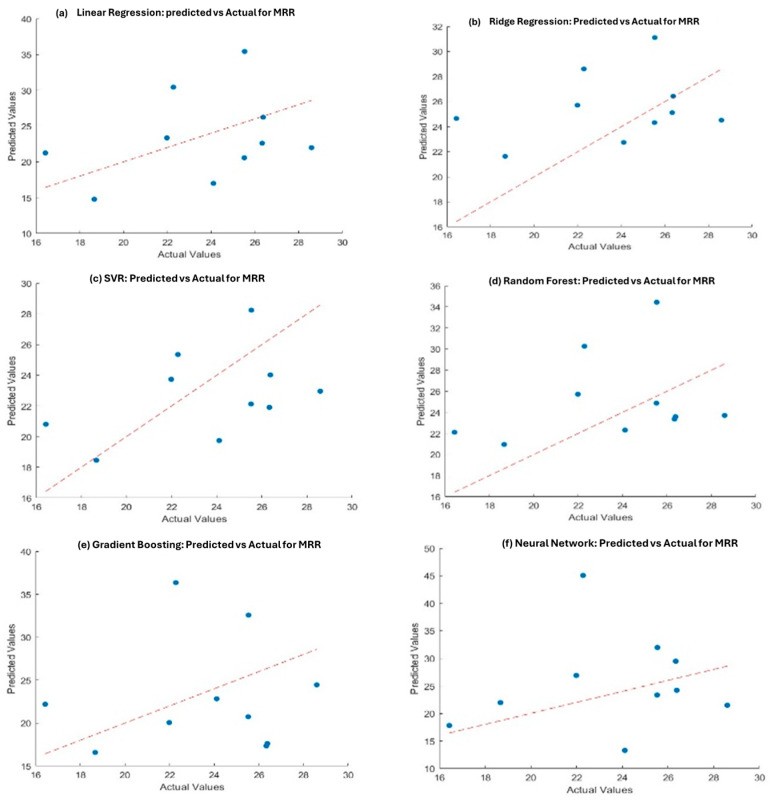
Actual vs. predicted values for MRR in different models, including (**a**) Linear Regression, (**b**) Ridge Regression, (**c**) Support Vector Regression (SVR), (**d**) Random Forest, (**e**) Gradient Boosting, and (**f**) Neural Networks.

Table 5 summarizes the model performance for predicting MRR and EWR. Gradient Boosting outperformed other models in predicting MRR, showing the lowest MSE (28.28) and RMSE (5.31) with a relatively high R^2^ value (0.54) as seen in Figure 12. Neural Networks, while slightly less accurate in MRR, performed best for EWR, achieving the highest R^2^ (0.87) and lowest MSE (7.58).

**Figure 13:**
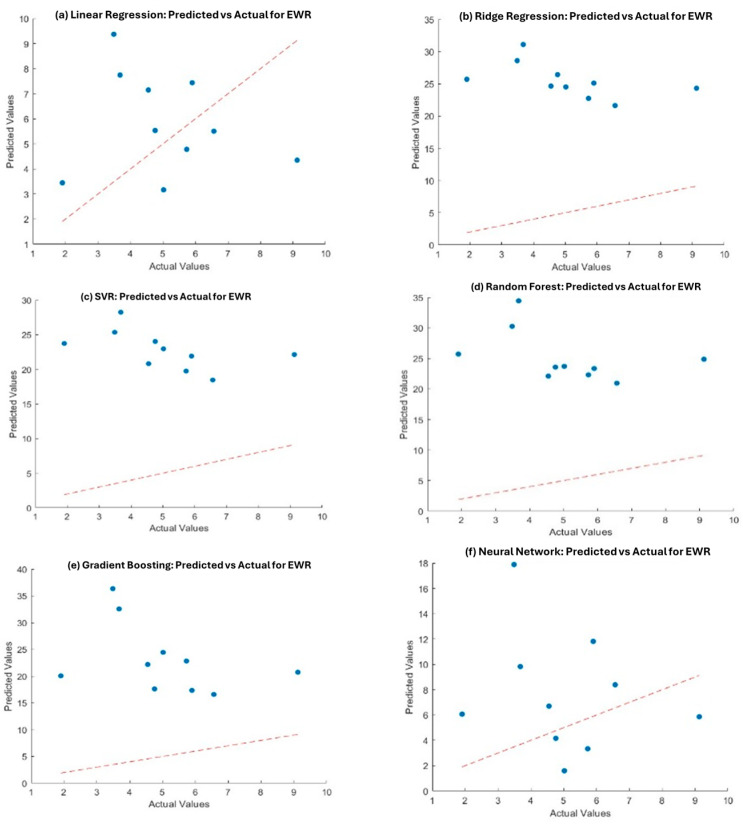
Actual vs. predicted values for EWR in different models, including (**a**) Linear Regression, (**b**) Ridge Regression, (**c**) Support Vector Regression (SVR), (**d**) Random Forest, (**e**) Gradient Boosting, and (**f**) Neural Networks.

Furthermore, support vector regression (SVR) exhibited poor performance, with negative R^2^ values for both MRR and EWR, indicating that it is not suitable for this dataset. Figure 13 shows the actual vs. predicted values for all six models used for EWR.

#### 3.3.2. Model Performance Metrics for Ra, Rq, and Rz

Table 6 presents the performance metrics for predicting the surface roughness parameters Ra (average roughness), Rq (root mean square roughness), and Rz (mean peak-to-valley height) using different machine learning models. Among all the models tested, Neural Network consistently emerged as the top performer, achieving high predictive accuracy across all surface parameters. For Ra, the Neural Network model achieved a remarkably low Mean Squared Error (MSE) of 1.12 and a high R^2^ value of 0.99, indicating that it could predict surface roughness with excellent precision. This model also demonstrated the lowest Absolute Mean Error (AME) and Sum of Squared Errors (SSE), outperforming all other models in predicting average surface finish.

Linear Regression, while significantly less accurate than the Neural Network, still performed reasonably well with an R^2^ of 0.76. However, Ridge Regression, Support Vector Regression (SVR), Random Forest, and Gradient Boosting models yielded negative R^2^ values, signifying poor predictive ability for Ra. These models tended to overfit or underfit the data, likely due to their limitations in modeling the nonlinear and complex interactions among EDM parameters. The trend continued with R_q_ predictions. Neural Networks again delivered superior results, achieving an MSE of 1.59 and an R^2^ of 0.98. The Linear Regression model produced an R^2^ of 0.81, making it the second-best performer, though it lagged significantly behind the Neural Network in terms of accuracy and consistency. The remaining models, including Ridge Regression, SVR, and Gradient Boosting, performed poorly, exhibiting high error values and negative R^2^ scores.

A similar pattern was observed for Rz predictions. Neural Network achieved the best performance with an MSE of 17.54 and an R^2^ of 0.90, which, while slightly lower than that for R_a_ and R_q_, still indicated strong predictive capacity. Linear Regression again provided moderate performance with an R^2^ of 0.74. The other models failed to generalize well, returning R^2^ values below zero, which highlights their unsuitability for predicting R_z_ under the current dataset and modeling configuration. The consistently strong performance of the Neural Network across all surface roughness metrics underscores its capability to model complex, nonlinear relationships between input parameters (current, voltage, and pulse-on time) and output responses. This performance advantage likely arises from its ability to learn intricate patterns and interactions within the data, which simpler models like Linear Regression or tree-based ensembles may fail to capture effectively. These findings affirm the suitability of Neural Networks for predictive modeling in EDM processes, particularly when the accurate forecasting of surface integrity is crucial for quality assurance and process optimization.

### 3.4. Analysis of Variance (ANOVA) with Pure EDM Oil and Al_2_O_3_-Infused EDM Oil

Table 7 presents the comparative analysis of machining performance using pure EDM oil versus Al_2_O_3_ nanoparticle-mixed EDM oil. The focus is on the following three primary responses: Material Removal Rate (MRR), Electrode Wear Rate (EWR), and Surface Roughness (Ra, Rq, Rz). The results indicate that the Al_2_O_3_ nanoparticle-mixed dielectric achieved higher MRR under optimal conditions, with improvements of approximately 15% over conventional EDM oil. This enhancement is attributed to improved thermal conductivity and better spark stability enabled by the presence of Al_2_O_3_ nanoparticles. EWR was consistently lower when using the nanoparticle-enhanced dielectric. At 30 A current and 75 µs pulse-on time, the EWR was reduced by around 20% compared to the conventional dielectric. This reduction highlights the improved cooling and debris removal capabilities of the Al_2_O_3_-mixed oil.

In terms of surface roughness, the nanoparticle dielectric achieved an average 10% improvement in Ra, Rq, and Rz values, indicating smoother surfaces. This is linked to enhanced spark energy dispersion and reduced arcing effects due to the dielectric’s superior insulation characteristics. Table 7 also includes ANOVA results identifying the significant parameters (*p* < 0.05). For MRR and EWR, both current and voltage were influential. Surface roughness parameters were primarily affected by current and pulse-on time. The R-squared values suggest a good model fit for EWR and Rz and a moderate fit for MRR and Ra.

### 3.5. Optical Microscopy and Surface Topography Analysis

Optical microscopy was used to evaluate the surface morphology of gunmetal workpieces before and after EDM machining under various dielectric conditions. Observations were made at magnifications of 5× and 10× to assess both macro and micro features of the surface.

**Figure 14:**
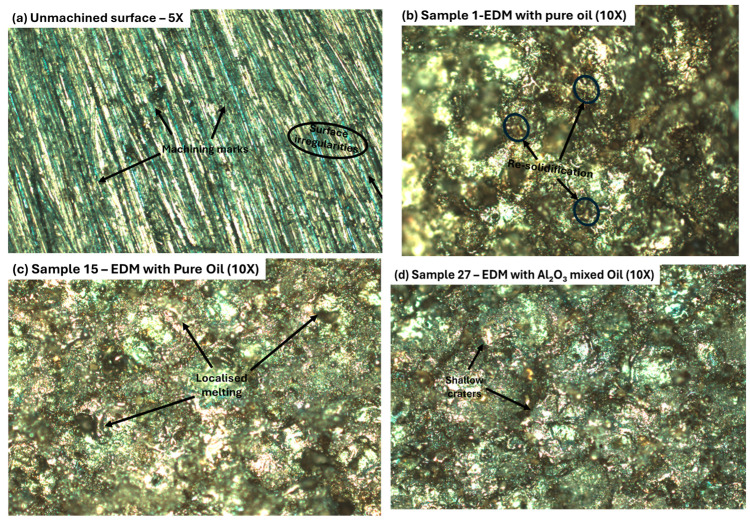
Optical microscopy images of gunmetal workpiece surfaces, i.e., (**a**) unmachined surface; (**b**) Sample 1; (**c**) Sample 15; and (**d**) Sample 27.

Figure 14a shows the unmachined gunmetal surface at 5× magnification, where clear machining marks, grooves, and irregularities can be seen. This serves as a baseline to understand how EDM affects the surface. Figure 14b–d shows Samples 1, 15, and 27, respectively, at 10× magnification. Sample 1, machined with pure EDM oil at lower energy settings, shows some re-solidification and debris, with relatively minor surface damage. Sample 15, also machined with pure EDM oil but at higher energy, displays more melting, re-solidified layers, and some visible micro-cracks and pits. Sample 27, processed using Al_2_O_3_ nanoparticle-mixed EDM oil, shows a smoother and more consistent surface compared to the others. It has fewer defects, a thinner re-solidified layer, and almost no micro-cracks. This suggests that the nanoparticle-enhanced dielectric fluid improves heat dissipation and flushing, leading to better machining quality and indicating better surface finish with lower Ra, Rq, and Rz values.

**Figure 15:**
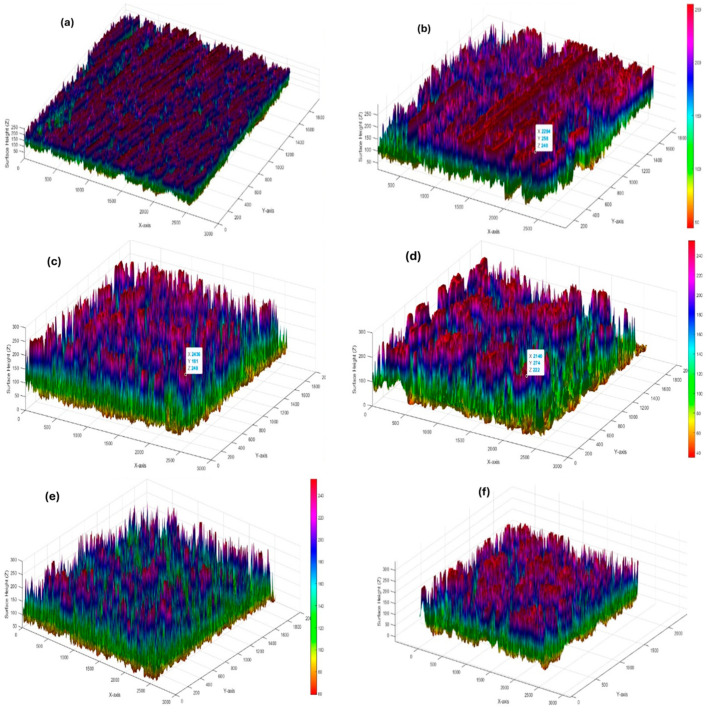
Three-dimensional surface topography of gunmetal workpieces. (**a**) Unmachined surface—5×, (**b**) unmachined surface—10×, (**c**) Sample 1—EDM with pure oil—5×, (**d**) Sample 1—EDM with pure oil—10×, (**e**) Sample 15—EDM with pure oil—10×, (**f**) Sample 27—EDM with Al_2_O_3_ oil—10×.

Figure 15 shows the 3D surface topography of the same samples at both 5× and 10× magnification. The unmachined sample (Figure 15a,b) has visible tool marks and a rough surface with uneven peaks and valleys. Sample 1 (Figure 15c,d), machined with pure EDM oil, shows some melted areas and solidified material on the surface, with moderate height variations. Sample 15 (Figure 15e), also machined with pure oil but at higher energy, appears rougher, with deeper valleys and more pronounced peaks. In contrast, Sample 27 (Figure 15f), processed with Al_2_O_3_-mixed EDM oil, has a noticeably flatter and smoother surface. The variations in height are reduced, suggesting improved surface quality. This reinforces the earlier findings that the nanoparticle-mixed dielectric helps achieve better machining results. Overall, the 3D topography images visually confirm the benefits of using Al_2_O_3_ in improving EDM surface characteristics.

The comparison of both magnifications provides a comprehensive understanding of the surface characteristics of the machined gunmetal. These observations are crucial for optimizing the machining process, as they help in correlating the machining parameters with the observed surface quality. Figure 15 shows the detailed surface topography analysis that aids in identifying potential issues that could affect the performance of the machined components in practical applications.

## 4. Conclusions

This study presented a comparative evaluation of conventional EDM oil and Al_2_O_3_ nanoparticle-mixed EDM oil for machining gunmetal using copper electrodes. The performance was assessed in terms of Material Removal Rate (MRR), Electrode Wear Rate (EWR), and surface roughness parameters (Ra, Rq, Rz), supported by predictive modeling through machine learning techniques. The experimental results clearly demonstrate the superiority of nanoparticle-enhanced dielectrics, while the machine learning models provide an effective framework for process optimization.

Key findings include the following:

- Productivity improvement: Al_2_O_3_ nanoparticle-mixed EDM oil achieved up to 15% higher MRR than conventional dielectric fluid under optimal conditions, highlighting its potential for rough machining applications where efficiency is critical.
- Tool life extension: EWR was reduced by ~20% with the Al_2_O_3_-based dielectric, indicating more efficient heat dissipation and reduced electrode degradation.
- Surface quality enhancement: Average surface roughness values improved by ~10% due to more stable discharge conditions and uniform flushing in the nanoparticle fluid.
- Microscopic evidence of new science: Surface analysis confirmed fewer micro-cracks and improved surface integrity when using the Al_2_O_3_-based dielectric, offering a clear scientific contribution to understanding dielectric effects on surface morphology.
- Predictive accuracy: Among the six machine learning models tested, Neural Networks and Gradient Boosting consistently delivered the highest prediction accuracy for MRR and surface finish, even with limited experimental data. This demonstrates the potential of combining data-driven approaches with EDM research.

While the fixed nanoparticle concentration (0.5 wt.%) ensured consistency, it also represents a limitation. Future research should perform a sensitivity analysis across varying nanoparticle concentrations to identify the optimal range as well as investigate long-term stability and applicability to other alloys. Comparative studies using different nanoparticles and advanced dielectric systems could further enhance the industrial scope of this work.

In summary, this research establishes that integrating nanoparticle-enhanced dielectrics with machine learning-driven process optimization not only improves productivity, tool life, and surface quality, but it also advances the scientific understanding of EDM processes.

## Figures and Tables

**Table 1:** Properties of electrode, workpiece, and machining parameters.

Material/Parameters	Properties and Its Values (Units)
Electrode	Copper (19 mm dia. and 82 mm length)
	Density—8.96 g/cm^3^
	Melting Point—1084 °C
	Electrical Conductivity—100% IACS
	Tensile Strength—200–250 MPa
	Thermal Conductivity—390–400 W/m·K
	Hardness (Vickers)—50–100 HV
Workpiece	Gunmetal (80 mm dia. and 10 mm thickness)
	Density—8.7 g/cm^3^
	Melting Point—1000–1050 °C
	Electrical Conductivity—15–18% IACS
	Tensile Strength—200–250 MPa
	Thermal Conductivity—40–50 W/m·K
	Hardness (Brinell)—60–100 HB
Nanoparticle	Al_2_O_3_ (Spherical with dia. 20–50 nm)
	Density—3.95–4.1 g/cm^3^
	Melting Point—2072 °C
	Electrical Resistivity—10^14^–10^16^ Ω·cm
	Thermal Conductivity—30 W/m·K
	Hardness (Mohs)—9
Dielectric	Pure EDM oil and Al_2_O_3_ mixed EDM oil
Current (I)	5, 10, and 15 A
Voltage (V)	30, 40, and 50 V
Pulse on time (T_on_)	30, 50, and 75 µs

**Table 2:** FTIR spectrum analysis with and without the addition of Al_2_O_3_ in EDM oil.

Wavenumber (cm^−1^)	Functional Group	EDM Oil (Sample 1)	EDM Oil + Al_2_O_3_ (Sample 2)
2900–2950	C-H Stretching (Alkanes)	Present	Present, with potential intensity changes
1750–1700	C=O Stretching (Carbonyls)	Possibly present	Possibly present with slight shifts
1450–1375	C-H Bending (Alkanes)	Present	Present, possibly modified
1000–1300	C-O Stretching (Alcohols, Ethers)	Present	Present with potential shifts
500–800	M-O (Metal–Oxygen) Stretching	Not Present	Present (due to Al_2_O_3_)

**Table 3:** Experimental design matrix for MRR, EWR, and surface roughness (Ra, Rq, Rz) for EDM oil.

Exp. No.	Current (A)	Voltage (V)	Ton (µs)	MRR (mg/min)	EWR (mg/min)	Ra (µm)	Rq (µm)	Rz (µm)
1	5	30	30	22.28 ± 0.45	3.49 ± 0.07	4.09 ± 0.08	5.02 ± 0.10	22.36 ± 0.45
2	5	30	50	40.72 ± 0.81	8.27 ± 0.17	4.52 ± 0.09	5.77 ± 0.12	29.25 ± 0.58
3	5	30	75	25.54 ± 0.51	3.68 ± 0.07	4.29 ± 0.09	5.26 ± 0.11	22.65 ± 0.45
4	5	40	30	20.28 ± 0.41	3.72 ± 0.07	3.51 ± 0.07	4.41 ± 0.09	20.93 ± 0.42
5	5	40	50	24.09 ± 0.48	4.40 ± 0.09	3.91 ± 0.08	4.91 ± 0.10	23.95 ± 0.48
6	5	40	75	26.75 ± 0.54	2.86 ± 0.06	4.27 ± 0.09	5.32 ± 0.11	25.51 ± 0.51
7	5	50	30	27.01 ± 0.54	6.24 ± 0.12	3.98 ± 0.08	4.96 ± 0.10	23.66 ± 0.47
8	5	50	50	23.26 ± 0.47	4.23 ± 0.08	4.48 ± 0.09	5.63 ± 0.11	27.95 ± 0.56
9	5	50	75	24.67 ± 0.49	3.77 ± 0.08	5.23 ± 0.10	6.26 ± 0.13	27.59 ± 0.55
10	10	30	30	31.59 ± 0.63	16.40 ± 0.33	4.34 ± 0.09	5.47 ± 0.11	26.76 ± 0.54
11	10	30	50	34.64 ± 0.69	16.71 ± 0.33	5.08 ± 0.10	6.16 ± 0.12	26.55 ± 0.53
12	10	30	75	47.10 ± 0.94	12.90 ± 0.26	5.10 ± 0.10	6.07 ± 0.12	26.27 ± 0.53
13	10	40	30	26.34 ± 0.53	5.90 ± 0.12	4.25 ± 0.09	5.25 ± 0.11	23.30 ± 0.47
14	10	40	50	26.77 ± 0.54	6.16 ± 0.12	5.82 ± 0.12	6.88 ± 0.14	28.78 ± 0.58
15	10	40	75	23.53 ± 0.47	3.82 ± 0.08	6.70 ± 0.13	8.01 ± 0.16	33.86 ± 0.68
16	10	50	30	12.58 ± 0.25	3.76 ± 0.08	4.88 ± 0.10	5.92 ± 0.12	26.69 ± 0.53
17	10	50	50	25.52 ± 0.51	9.13 ± 0.18	5.51 ± 0.11	7.01 ± 0.14	33.83 ± 0.68
18	10	50	75	21.99 ± 0.44	1.91 ± 0.04	5.07 ± 0.10	6.38 ± 0.13	30.34 ± 0.61
19	15	30	30	20.06 ± 0.40	7.25 ± 0.15	6.60 ± 0.13	8.26 ± 0.17	36.78 ± 0.74
20	15	30	50	20.63 ± 0.41	6.30 ± 0.13	5.06 ± 0.10	6.50 ± 0.13	30.61 ± 0.61
21	15	30	75	20.16 ± 0.40	6.78 ± 0.14	5.59 ± 0.11	6.85 ± 0.14	31.00 ± 0.62
22	15	40	30	16.96 ± 0.34	6.09 ± 0.12	4.55 ± 0.09	5.61 ± 0.11	25.44 ± 0.51
23	15	40	50	16.43 ± 0.33	4.55 ± 0.09	5.21 ± 0.10	6.84 ± 0.14	34.17 ± 0.68
24	15	40	75	23.98 ± 0.48	5.01 ± 0.10	5.38 ± 0.11	6.74 ± 0.13	32.59 ± 0.65
25	15	50	30	18.67 ± 0.37	6.57 ± 0.13	4.22 ± 0.08	5.52 ± 0.11	28.30 ± 0.57
26	15	50	50	24.11 ± 0.48	5.73 ± 0.11	4.68 ± 0.09	5.98 ± 0.12	28.77 ± 0.58
27	15	50	75	25.17 ± 0.50	5.28 ± 0.11	6.20 ± 0.12	7.77 ± 0.16	33.33 ± 0.67

**Table 4:** Experimental results for MRR, EWR, and surface roughness (Ra, Rq, Rz) for EDM oil mixed with Al_2_O_3_ nanoparticles.

Exp. No.	Current (A)	Voltage (V)	Ton (µs)	MRR (mg/min)	EWR (mg/min)	Ra (µm)	Rq (µm)	Rz (µm)
1	5	30	30	25.78 ± 0.52	3.52 ± 0.07	3.75 ± 0.08	4.60 ± 0.09	20.19 ± 0.40
2	5	30	50	36.79 ± 0.74	7.47 ± 0.15	4.14 ± 0.08	5.36 ± 0.11	27.71 ± 0.55
3	5	30	75	28.62 ± 0.57	3.32 ± 0.07	3.93 ± 0.08	4.88 ± 0.10	21.07 ± 0.42
4	5	40	30	22.52 ± 0.45	3.54 ± 0.07	3.26 ± 0.07	4.10 ± 0.08	19.36 ± 0.39
5	5	40	50	26.76 ± 0.54	4.18 ± 0.08	3.64 ± 0.07	4.57 ± 0.09	22.07 ± 0.44
6	5	40	75	32.43 ± 0.65	2.31 ± 0.05	3.97 ± 0.08	4.65 ± 0.09	23.72 ± 0.47
7	5	50	30	28.71 ± 0.57	5.85 ± 0.12	3.70 ± 0.07	4.59 ± 0.09	22.61 ± 0.45
8	5	50	50	25.60 ± 0.51	3.98 ± 0.08	4.17 ± 0.08	5.12 ± 0.10	23.50 ± 0.47
9	5	50	75	27.22 ± 0.54	3.58 ± 0.07	4.86 ± 0.10	5.52 ± 0.11	25.66 ± 0.51
10	10	30	30	34.75 ± 0.70	15.38 ± 0.31	4.04 ± 0.08	5.10 ± 0.10	24.89 ± 0.50
11	10	30	50	38.11 ± 0.76	15.86 ± 0.32	4.72 ± 0.09	5.75 ± 0.12	24.68 ± 0.49
12	10	30	75	51.81 ± 1.04	12.26 ± 0.25	4.74 ± 0.09	5.67 ± 0.11	21.53 ± 0.43
13	10	40	30	28.97 ± 0.58	5.60 ± 0.11	3.95 ± 0.08	4.88 ± 0.10	22.67 ± 0.45
14	10	40	50	29.45 ± 0.59	5.85 ± 0.12	5.41 ± 0.11	6.40 ± 0.13	26.77 ± 0.54
15	10	40	75	25.88 ± 0.52	3.93 ± 0.08	6.23 ± 0.12	7.15 ± 0.14	31.48 ± 0.63
16	10	50	30	20.84 ± 0.42	3.57 ± 0.07	4.53 ± 0.09	5.51 ± 0.11	24.82 ± 0.50
17	10	50	50	28.08 ± 0.56	8.67 ± 0.17	5.12 ± 0.10	6.51 ± 0.13	31.16 ± 0.62
18	10	50	75	24.81 ± 0.50	1.81 ± 0.04	4.71 ± 0.09	5.94 ± 0.12	28.21 ± 0.56
19	15	30	30	22.07 ± 0.44	6.89 ± 0.14	6.14 ± 0.12	7.68 ± 0.15	32.40 ± 0.65
20	15	30	50	22.99 ± 0.46	5.99 ± 0.12	4.70 ± 0.09	5.04 ± 0.10	28.47 ± 0.57
21	15	30	75	22.19 ± 0.44	6.44 ± 0.13	5.19 ± 0.10	6.37 ± 0.13	28.83 ± 0.58
22	15	40	30	19.66 ± 0.39	5.79 ± 0.12	4.23 ± 0.08	5.22 ± 0.10	23.66 ± 0.47
23	15	40	50	18.07 ± 0.36	4.32 ± 0.09	3.85 ± 0.08	6.36 ± 0.13	31.78 ± 0.64
24	15	40	75	26.38 ± 0.53	4.76 ± 0.10	5.00 ± 0.10	6.27 ± 0.13	30.31 ± 0.61
25	15	50	30	20.54 ± 0.41	6.24 ± 0.12	3.92 ± 0.08	5.13 ± 0.10	26.32 ± 0.53
26	15	50	50	24.52 ± 0.49	5.44 ± 0.11	4.35 ± 0.09	5.56 ± 0.11	26.76 ± 0.54
27	15	50	75	28.59 ± 0.57	5.02 ± 0.10	5.77 ± 0.12	7.23 ± 0.14	30.99 ± 0.62

**Table 5:** Model performance metrics for MRR and EWR.

**Performance Metrics for MRR**					
**Model**	**MSE (MRR)**	**AME (MRR)**	**SSE (MRR)**	**RMSE (MRR)**	**R^2^ (MRR)**
Linear Regression	57.60	6.34	576.04	7.58	0.069
Ridge Regression	52.87	5.15	528.74	7.27	0.15
SVR	68.98	5.90	689.81	8.30	−0.12
Random Forest	42.90	4.82	429.05	6.55	0.30
Gradient Boosting	28.28	4.36	282.82	5.31	0.54
Neural Network	68.96	7.18	689.69	8.30	0.65
**Performance Metrics for EWR**					
**Model**	**MSE (EWR)**	**AME (EWR)**	**SSE (EWR)**	**RMSE (EWR)**	**R^2^ (EWR)**
Linear Regression	12.17	2.45	121.73	3.49	0.2390
Ridge Regression	353.81	18.43	3538.072	18.81	−21.1177
SVR	282.37	16.09	2823.67	16.80	−16.6517
Random Forest	334.48	17.99	3344.77	18.29	−19.9093
Gradient Boosting	388.93	18.97	3889.26	19.72	−23.3131
Neural Network	7.58	1.99	75.83	2.75	0.8726

**Table 6:** Model performance metrics for R_a_, R_q_, and R_z_.

**Performance Metrics for R_a_**					
**Model**	**MSE (R**_a_ **)**	**AME (R**_a_ **)**	**SSE (R**_a_ **)**	**RMSE (R**_a_ **)**	**R^2^ (R**_a_ **)**
Linear Regression	0.17	0.37	1.66	0.41	0.76
Ridge Regression	434.62	20.76	4346.21	20.85	−629.42
SVR	346.72	18.42	3467.15	18.62	−501.91
Random Forest	418.85	20.32	4188.50	20.47	−606.54
Gradient Boosting	482.05	21.30	4820.48	21.96	−698.21
Neural Network	1.120	0.92	11.95	1.09	0.99
**Performance Metrics for R_q_**					
**Model**	**MSE (R_q_)**	**AME (R_q_)**	**SSE (R_q_)**	**RMSE (R_q_)**	**R^2^ (R_q_)**
Linear Regression	0.21	0.40	2.14	0.46	0. 81
Ridge Regression	390.22	19.63	3902.17	19.75	−354.67
SVR	308.13	17.30	3081.26	17.55	−279.85
Random Forest	375.56	19.20	3755.58	19.38	−341.31
Gradient Boosting	436.45	20.18	4364.47	20.89	−396.81
Neural Network	1.59	1.05	15.94	1.26	0.98
**Performance Metrics for R_z_**					
**Model**	**MSE (R_Z_)**	**AME (R_Z_)**	**SSE (R_Z_)**	**RMSE (R_Z_)**	**R^2^ (R_Z_)**
Linear Regression	5.24	1.88	52.43	2.29	0.74
Ridge Regression	30.84	4.46	308.42	5.55	−0.51
SVR	53.79	6.05	537.88	7.33	−1.63
Random Forest	35.41	4.67	354.06	5.95	−0.73
Gradient Boosting	55.51	6.08	555.08	7.45	−1.72
Neural Network	17.54	3.39	175.36	4.19	0.90

**Table 7:** Analysis of variance for MRR, EWR, and surface roughness parameters (R_a_, R_q_, and R_z_).

Response	Significant Factors (*p* < 0.05)	R^2^	R^2^ (adj)	R^2^ (pred)	Interpretation
MRR	Current (A), Voltage (V)	47.50%	31.74%	4.31%	Moderate model fit; both current and voltage significantly affect MRR.
EWR	Current (A), Voltage (V)	56.58%	43.56%	20.87%	Good model fit; both current and voltage significantly affect EWR.
Ra	Current (A), Ton (µs)	52.77%	38.61%	13.93%	Moderate model fit; current and Ton significantly affect Ra.
Rq	Current (A), Ton (µs)	55.48%	42.12%	18.85%	Good model fit; current significantly affects Rq.
Rz	Current (A)	57.15%	44.30%	21.91%	Good model fit; current significantly affects Rz.
MRR	Current (A), Voltage (V)	47.50%	31.74%	4.31%	Moderate model fit; both current and voltage significantly affect MRR.
EWR	Current (A), Voltage (V)	56.58%	43.56%	20.87%	Good model fit; both current and voltage significantly affect EWR.
Ra	Current (A), Ton (µs)	52.77%	38.61%	13.93%	Moderate model fit; current and Ton significantly affect Ra.
Rq	Current (A), Ton (µs)	55.48%	42.12%	18.85%	Good model fit; current significantly affects Rq.
Rz	Current (A)	57.15%	44.30%	21.91%	Good model fit; current significantly affects Rz.

## Data Availability

The original contributions presented in this study are included in the article. Further inquiries can be directed to the corresponding authors.

## References

[1] Kumar A., Mandal A., Dixit A.R., Das A.K. (2018). Performance evaluation of Al2O3 nano powder mixed dielectric for electric discharge machining of Inconel 825. Mater. Manuf. Process..

[2] Mohanty S., Mishra A., Nanda B.K., Routara B.C. (2018). Multi-objective parametric optimization of nano powder mixed electrical discharge machining of AlSiCp using response surface methodology and particle swarm optimization. Alex. Eng. J..

[3] Kumar D., Singh N.K., Bajpai V. (2020). Recent trends, opportunities and other aspects of micro-EDM for advanced manufacturing: A comprehensive review. J. Braz. Soc. Mech. Sci. Eng..

[4] Kumar A., Mandal A., Dixit A.R., Das A.K., Kumar S., Ranjan R. (2019). Comparison in the performance of EDM and NPMEDM using Al_2_O_3_ nanopowder as an impurity in DI water dielectric. Int. J. Adv. Manuf. Technol..

[5] Niu Y.J., Hong W. (2019). Dielectric nanomaterials for power energy storage: Surface modification and characterization. ACS Appl. Nano Mater..

[6] Venkatesh M.S., Raghavan G.S.V. (2005). An overview of dielectric properties measuring techniques. Can. Biosyst. Eng..

[7] Li Y., Luo Y., Deng H., Shi S., Tian S., Wu H., Tang J., Zhang C., Zhang X., Zha J. (2024). Advanced dielectric materials for triboelectric nanogenerators: Principles, methods, and applications. Adv. Mater..

[8] Wu K., Lei C., Yang W., Chai S., Chen F., Fu Q. (2016). Surface modification of boron nitride by reduced graphene oxide for preparation of dielectric material with enhanced dielectric constant and well-suppressed dielectric loss. Compos. Sci. Technol..

[9] Niu Q., Luo J., Xia Y., Sun S., Chen Q. (2017). Surface modification of bio-char by dielectric barrier discharge plasma for Hg0 removal. Fuel Process. Technol..

[10] Mohanty S., Routara B.C., Nanda B.K., Das D.K., Sahoo A.K. (2018). Study of machining characteristics of Al-SiCp12% composite in nano powder mixed dielectric electrical discharge machining using RSM. Mater Today Proc..

[11] Rai N., Singh C.P., Ranjta L. (2022). Structural, Thermal and Electrical Studies of Al_2_O_3_ Nanoparticle Soaked Electrolyte Gel Films for Novel Proton Conducting (H^+^ Ion) Eco-Friendly Device Applications. Am. J. Nano Res. Appl..

[12] Hazra S., Mohanty S., Kumar S., Basak R., Das A.K. (2022). Experimental investigation of powder mixed micro-electrical discharge drilling on SS304 substrate. Mater. Today Proc..

[13] Nowicki R., Oniszczuk-Świercz D., Świercz R. (2024). Experimental Investigation on the Impact of Graphite Electrodes Grain Size on Technological Parameters and Surface Texture of Hastelloy C-22 after Electrical Discharge Machining with Negative Polarity. Materials.

[14] Makenzi M.M., Bernard W.I. A review of flushing techniques used in electrical discharge machining. Proceedings of the Sustainable Research and Innovation Conference.

[15] Wong Y.S., Lim L.C., Lee L.C. (1995). Effects of flushing on electro-discharge machined surfaces. J. Mater. Process. Technol..

[16] Masuzawa T., Heuvelman C. (1983). A self-flushing method with spark-erosion machining. CIRP Ann..

[17] Yadav S., Agarwal D., Sharma A.K., Singh R.K., Chauhan S., Mohanty S. (2025). Experimental investigation of Al_2_O_3_ nano-powder-mixed dielectric in EDM-assisted micro-milling. Micromachines.

[18] Patel Gowdru Chandrashekarappa M., Kumar S., Pimenov D.Y., Giasin K. (2021). Experimental analysis and optimization of EDM parameters on HcHcr steel in context with different electrodes and dielectric fluids using hybrid taguchi-based PCA-utility and CRITIC-utility approaches. Metals.

[19] Jv R., Abimannan G. (2022). Analysis on the performance of micro and nano molybdenum di-sulphide powder suspended dielectric in the electrical discharge machining process—A comparison. Nanomaterials.

[20] Yadav S., Kumar V., Misra J.P., Singh R.K., Upadhyay V. (2023). Surface modification by electrical discharge machining: A systematic literature review and bibliometric analysis. Proc. Inst. Mech. Eng. Part E J. Process. Mech. Eng..

[21] Boominathan E., Krishnan G., Gurijala C., Vm J. (2024). Studies on the effect of SiC nanopowder concentration and discharge energy on surface roughness and recast layer in micro ED milling of Inconel 718 alloy. Phys. Script..

[22] Chaurasia S., Bisaria H., Singh S., Debnath K. (2024). Powder mixed micro-electric discharge milling of Ni-rich NiTi SMA: An investigation on machining performance and biocompatibility. Mater. Manuf. Process..

[23] Mahajan R., Krishna H., Singh A.K., Ghadai R.K. (2018). A review on copper and its alloys used as electrode in EDM. IOP Conference Series: Materials Science and Engineering.

[24] Li L., Wong Y.S., Fuh J.Y.H., Lu L. (2001). EDM performance of TiC/copper-based sintered electrodes. Mater. Des..

[25] Balasubramanian P., Senthilvelan T. (2014). Optimization of machining parameters in EDM process using cast and sintered copper electrodes. Procedia Mater. Sci..

[26] Khan A.A. (2008). Electrode wear and material removal rate during EDM of aluminum and mild steel using copper and brass electrodes. Int. J. Adv. Manuf. Technol..

[27] Gaitonde V.N., Karnik S.R., Faustino M., Davim J.P. (2010). Machinability analysis in turning tungsten–copper composite for application in EDM electrodes. Int. J. Refract. Met. Hard Mater..

[28] Sharma D., Hiremath S.S. (2021). Review on tools and tool wear in EDM. Mach. Sci. Technol..

[29] Kossymbayev A., Ali S., Talamona D., Perveen A. (2025). Powder-Mixed Micro Electrical Discharge Machining-Assisted Surface Modification of Ti-35Nb-7Zr-5Ta Alloy in Biomedical Applications. Eng. Proc..

[30] Kaigude A.R., Khedkar N.K., Jatti V.S., Salunkhe S., Cep R., Nasr E.A. (2024). Surface roughness prediction of AISI D2 tool steel during powder mixed EDM using supervised machine learning. Sci. Rep..

[31] Gupta A., Sinha A., Jain S. (2019). Effect of Al_2_O_3_ Nanoparticles on Performance of EDM Process Using Oil-Based Dielectrics. J. Manuf. Process..

[32] Hussain A., Mahmud M. (2017). Influence of Deionized Water on Performance of EDM Process: A Comparative Study. Int. J. Adv. Manuf. Technol..

[33] Khan M., Patel R. (2022). Nanoparticle-Enhanced Dielectrics in EDM: A Review of Recent Advancements. Mater. Sci. Eng. R. Rep..

[34] Kumar A., Rajurkar K.P. (2016). Comparative Study of EDM Performance with Conventional and Hybrid Dielectrics. J. Manuf. Sci. Eng..

[35] Pramanik A., Ghosh A. (2014). Effect of Dielectric Fluids on EDM Performance in Machining of Titanium Alloy. Procedia CIRP.

[36] Singh S., Kumar V., Gupta V. (2015). Influence of Deionized Water on EDM Performance: A Review. Mater. Manuf. Process..

[37] Soni S., Kumar M. (2018). Study on the Impact of EDM Oil on Machining Efficiency and Surface Integrity. Precis. Eng..

[38] Jameson E.C. (2001). Electrical Discharge Machining.

[39] Saxena K.K., Agarwal S., Mukhopadhyay J. Effect of Machining Parameters on Surface Roughness in µ-EDM of Conductive SiC. Proceedings of the ASME 2014 International Mechanical Engineering Congress and Exposition, Volume 2B: Advanced Manufacturing.

[40] Singh B., Kumar J., Kumar S. (2014). Experimental Investigation on Surface Characteristics in Powder-Mixed Electro Discharge Machining of AA6061/10% SiC Composite. Mater. Manuf. Process..

[41] Yadav A., Mohanty S., Dwivedi S., Dixit A.R. (2022). Selective surface modification of SS304 using hybrid powder-mixed EDC process. Surf. Eng..

[42] Kiran P., Mohanty S., Das A.K. (2022). Sustainable surface modification of Ti-alloy using powder mixed in bio-dielectrics through micro-electrical discharge coating process. J. Clean. Prod..

[43] Tyagi R., Mandal A., Das A.K., Tripathi A., Prakash C., Campilho R., Saxena K.K. (2022). Electrical discharge coating a potential surface engineering technique: A state of the art. Processes.

[44] Mazarbhuiya R.M., Rahang M., Saha S., Veeman D. (2025). Parametric study on selective surface modification in EDM using TOPSIS. Surf. Eng..

[45] Singh D., Goyal P., Sehgal S. (2024). The Machining and Surface Modification of H13 Die Steel via the Electrical Discharge Machining Process Using Graphite Mixed Dielectric. J. Manuf. Mater. Process..

